# Dr. John M. Farrington

**Published:** 1917-06

**Authors:** 


**Dr. John M. Farrington,** N. Y. Med. College, 1857, died at his home in Binghamton, April 18, aged 84.

